# Enhancing sugarcane’s drought resilience: the influence of Streptomycetales and Rhizobiales

**DOI:** 10.3389/fpls.2024.1471044

**Published:** 2024-11-29

**Authors:** Mianhe Chen, Yuanjun Xing, Chunyi Chen, Ziting Wang

**Affiliations:** State Key Lab for Conservation and Utilization of Subtropical Agri-Biological Resources, Guangxi Key Lab for Sugarcane Biology, College of Agriculture, Guangxi University, Nanning, China

**Keywords:** sugarcane, rhizosphere bacterial community, drought tolerance, Streptomycetales, Rhizobiales, plant growth-promoting bacteria (PGPB)

## Abstract

Drought stress is a critical environmental factor affecting sugarcane yield, and the adaptability of the sugarcane rhizosphere bacterial community is essential for drought tolerance. This review examines the adaptive responses of sugarcane rhizosphere bacterial communities to water stress and explores their significant role in enhancing sugarcane drought tolerance. Under drought conditions, the sugarcane rhizosphere bacterial community undergoes structural and functional shifts, particularly the enrichment of beneficial bacteria, including Streptomycetales and Rhizobiales. These bacteria enhance sugarcane resilience to drought through various means, including nutrient acquisition and phytohormone synthesis. Furthermore, changes in the rhizosphere bacterial community were closely associated with the composition and levels of soil metabolites, which significantly influenced the physiological and biochemical processes of sugarcane during drought stress. This study deepens our understanding of rhizosphere bacterial communities and their interactions with sugarcane, laying a scientific foundation for developing drought-resistant sugarcane varieties, optimizing agricultural practices, and opening new avenues for agricultural applications.

## 1 Introduction

Sugarcane (*Saccharum officinarum* L.) cultivation is crucial for both sugar and bioenergy production. However, this cultivation faces significant challenges that can severely affect growth and yield, particularly in areas where drought is prevalent (Ferreira et al., 2017; Dlamini, 2021). Microorganisms are emerging as pivotal agents in mitigating the adverse effects of climate change, notably through their capacity to ameliorate the challenges posed by abiotic stressors, including water scarcity, which plants frequently encounter (Zhang et al., 2022). Fungi, including genera such as *Trichoderma* and essential arbuscular mycorrhizal fungi, along with bacteria such as *Azospirillum*, *Bacillus*, and *Pseudomonas*, constitute a vital component of the Earth’s microbial community. Their multifaceted functions in the natural world include decomposing organic matter, mediating the nitrogen cycle, and enhancing plant growth. These microorganisms contribute not only to ecological balance but are also emerging as potential mitigators of climate change (Hu et al., 2022; Qin et al., 2024). Recent scientific insights have revealed that they may also serve as climate change adapters, with certain fungi and bacteria capable of sequestering atmospheric carbon, thereby augmenting soil carbon reserves and slowing the pace of global warming (Zhu et al., 2024). Moreover, by ameliorating soil structure and increasing soil water retention, these microbes enhance the ability of plants to withstand extreme climatic conditions, including drought, thereby mitigating the impacts of such environmental stresses (Ahmed et al., 2019). The resilience of sugarcane in the face of adversity is closely related to the dynamics of its rhizosphere bacterial communities (Liu et al., 2021a). These communities are gaining increasing recognition for their indispensable roles in enhancing plant health and productivity (Backer et al., 2018) (**Table 1**). These communities are not only passive inhabitants of the soil; they also actively interact with plant root systems, forming a network of symbiotic relationships (Liu et al., 2021b). They significantly aid plant adaptations to water scarcity by influencing soil nutrient cycling and modulating stress responses (Rolli et al., 2015; Vejan et al., 2016; Vurukonda et al., 2016). This interplay is crucial for plant survival and optimal performance under drought-prone conditions.

**Table 1:** Mechanisms employed by rhizosphere bacteria to ameliorate drought stress in plants.

Bacteria	Plants	Description	Reference
*Bacillus amyloliquefaciens*	*Mentha piperita* L.	Increased total phenol content, enhanced DPPH free radical scavenging, and reduced membrane lipid peroxidation	Chiappero et al. (2019)
*Bacillus amyloliquefaciens*	*Cicer arietinum* L.	Biofilm formation, produced siderophores, and reduced oxidative stress	Kumar et al. (2016)
*Bacillus rugosus*	*Triticum aestivum* L.	Biofilm formation, produced siderophores, and increased nitrogen fixation capacity	Salem et al. (2024)
*Bacillus subtilis*	*Saccharum officinarum* L.	Improved nutrient uptake, accumulated osmoregulatory compounds, and improved water use efficiency	Fonseca et al. (2022)
*Streptomyces albidoflavus* and *Streptomyces rochei*	*Avena nuda* L.	Removal of excess ROS, improved root morphology, and accumulated osmoregulatory compounds	Qiao et al. (2024)
*Streptomyces chartreusis* and *Streptomyces griseorubiginosus*	*Saccharum officinarum* L.	Production of indole-3-acetic acid (IAA), regulation of root morphology, and improvement of water use efficiency	Wang et al. (2018b; 2023c)
*Streptomyces pseudovenezuelae*	*Solanum lycopersicum*	Produced 1-aminocyclopropane-1-carboxylate (ACC) deaminase, increased total sugar content, regulated expression of transcription factors, and increased antioxidant enzyme activity	Abbasi et al. (2020)
*Arthrobacter arilaitensis* and *Streptomyces pseudovenezuelae*	*Zea mays* L.	Phytohormonal modifications, production of exopolysaccharides, and accumulation of osmolytes	Chukwuneme et al. (2020)
*Streptomyces pactum*	*Triticum aestivum* L.	Increased osmoregulatory compounds, produced ACC deaminase, reduced membrane damage, and increased efficiency of photosynthesis	Yang et al. (2024)
*Mesorhizobium huakuii*	*Astragalus sinicus* L.	Accumulated solutes and increased nitrogen fixation and ammonia assimilation	Liu et al. (2022b)
*Azospirillum* sp.	*Triticum aestivum* L.	Production of plant hormones and promotion of root elongation	Moutia et al. (2010)
*Rhizobium japonicum*, *Azotobacter chroococcum*, and *Azospirillum brasilense*	*Glycine max* (L.) Merr.	Reduced electrolyte leakage, increased nitrogen fixation, and ammonia assimilation	Abbasi et al. (2013)
*Sphingomonas* sp.	*Zea mays* L.	Regulated osmotic compounds and increased antioxidant enzyme activity	Wang et al. (2022)
*Sphingomonas* sp.	*Arabidopsis thaliana* (L.) Heynh.	Promoted growth of lateral roots and root hairs, influenced levels of plant hormones, and increased proline content	Luo et al. (2019)
*Sphingomonas panaciterrae*	*Spinacia oleracea* L.	Improved nutrient uptake and increased antioxidant and vitamin C content	Sultana et al. (2024)
*Rhizophagus* spp. and *Burkholderia seminalison*	*Lycopersicon esculatum* and *Capsicum annuum*	Increased antioxidant enzyme activity, improved root structure, improved water use efficiency, and enhanced colonization by mycorrhizal fungi	Tallapragada et al. (2016)
*Mitsuaria* sp. and *Burkholderia* sp.	*Zea mays* L.	Reduced membrane damage and increased antioxidant enzyme activity	Huang et al. (2017)
*Burkholderia* sp.	*Cucumis sativus* L.	Regulation of gene expression and increases proline synthesis	Wang et al. (2023a)
*Burkholderia* sp.	*Saccharum officinarum* L.	Regulation of carotenoid biosynthesis, terpenoid skeleton formation, starch and sucrose metabolism, and phytohormone signaling	Nong et al. (2023)
*Pseudomonas fluorescens*	*Catharanthus roseus* (L.) G. Don	Promoted root system development, produced phytohormones, and increased alkaloid content	Jaleel et al. (2007)
*Pseudomonas* sp.	*Andropogon gerardii*	Produced ACC deaminase and regulated nitrogen transformation gene expression	Sarkar et al. (2022)

Roots are essential for plants to absorb water from the soil. During drought, crop roots and rhizosphere soil microorganisms are the first to detect and respond to environmental changes. These rhizosphere microorganisms exhibit heightened sensitivity to changes in the soil environment and often respond more quickly than plants (Cai and Ahmed, 2022; Cai et al., 2022). Complex interactions among rhizosphere microorganisms are key factors in the rapid adaptation of plants to soil-borne environmental stresses (Giehl and von Wirén, 2014; Pascale et al., 2020). Ecological and evolutionary studies provide compelling evidence that, in the short to medium term, the resilience and productivity of plants in the face of global warming are likely to be significantly influenced by the intricate dynamics within host-associated microbiomes (Trivedi et al., 2022). In their quest to mitigate the adverse consequences of drought on agricultural productivity, researchers are pursuing a range of biotechnological strategies, from refining traditional breeding techniques to applying innovative genetic engineering to enhance sugarcane drought tolerance (Sugiharto, 2018; Misra et al., 2022). Rhizosphere bacteria are pivotal in the biotechnological exploration of drought tolerance in sugarcane. These microorganisms can significantly augment the adaptive capacity and resilience of plants under arid conditions through a spectrum of physiological and biochemical mechanisms (Chieb and Gachomo, 2023). In sugarcane breeding, understanding how various drought-tolerant root system architectures influence the rhizosphere microbial community is pivotal. This analysis not only underpins the theoretical framework for drought-tolerant breeding but also propels the development of new sugarcane varieties adapted to withstand arid conditions (Liu et al., 2021a). By integrating the selection of drought-resistant traits with an in-depth examination of rhizosphere microbial dynamics, breeders can develop novel sugarcane cultivars with superior adaptability to water-deficient environments. Similarly, the introduction of drought tolerance-related genes into the sugarcane genome can lead to the development of transgenic sugarcane varieties with enhanced drought tolerance (Kumar et al., 2024). For example, by expressing the betaine synthase gene in sugarcane, the accumulation of betaine is increased, and the osmoregulatory capacity of cells is improved, thereby enhancing the survival of sugarcane under drought conditions (Rahman et al., 2021). Moreover, the exploration of sugarcane drought resistance mechanisms through advanced gene editing techniques, including RNA interference (RNAi) or CRISPR/Cas9, targeting both transcription factors and genes with currently obscure functions, holds promise for further enhancement of these mechanisms (Mall et al., 2024). In addition, genetic engineering not only focuses on the genetic improvement of sugarcane itself but also involves the interaction of the rhizosphere bacterial community. Additionally, genetic engineering targets not only the enhancement of sugarcane genetic characteristics but also the interactions within the rhizosphere bacterial community. The alteration of the soil environment by transgenic sugarcane under drought stress conditions affects the composition of the rhizosphere bacterial community, which subsequently modulates the physiological responses of sugarcane (Zhao et al., 2020). These studies indicate that genetic engineering can enhance the drought tolerance of sugarcane through direct genetic intervention and concurrently bolster its adaptive capacity to drought stress indirectly by modulating the structure and functionality of the associated rhizosphere bacterial community.

**Figure 1:**
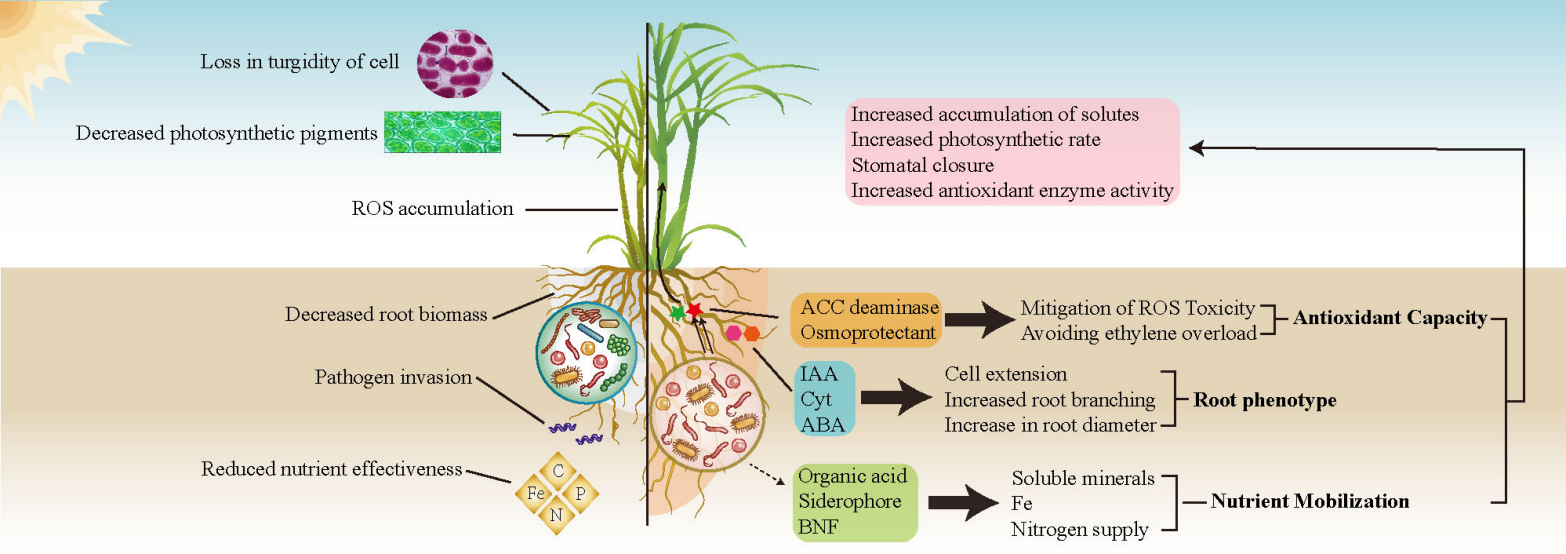
A comparison between water-deficient sugarcane without drought-resistant bacterial communities (left) and that with such communities (right). In the absence of a drought-resistant bacterial community, sugarcane is adversely affected, with reduced nutrient acquisition due to decreased water uptake, leading to stunted growth, an increase in reactive oxygen species (ROS), and impaired root development. Conversely, the recruitment of a drought-resistant bacterial community in sugarcane results in the production of essential phytohormones, including cytokinins (Cyt), indole-3-acetic acid (IAA), and abscisic acid (ABA). These phytohormones promote root growth and enhance the capacity for water and nutrient absorption by plants under drought stress. Furthermore, these beneficial bacteria also produce 1-aminocyclopropane-1-carboxylate (ACC) deaminase and osmoprotectants, which help to counteract the negative effects of ROS and mitigate membrane damage caused by drought conditions. Beyond their direct influence on sugarcane, these bacterial communities also increase soil nutrient availability through biological nitrogen fixation (BNF) and the secretion of secondary metabolites, including organic acids and iron-chelating compounds.

Variations in the diversity and abundance of sugarcane rhizosphere bacterial communities are pivotal in modulating soil nutrient availability and physiological responses of sugarcane to drought (Pereira et al., 2022) (**Figure 1**). Under drought stress, the structure and function of these bacterial communities undergo significant shifts and profoundly influence soil nutrient dynamics and plant interactions (Naylor and Coleman-Derr, 2018; Ling et al., 2022; Bandopadhyay et al., 2024). Particularly, under drought conditions, plants can alter the profile of soil metabolites in the rhizosphere via root exudation, effectively promoting the proliferation of specific bacteria capable of mitigating stress (Canarini and Dijkstra, 2015; Song et al., 2020). As a perennial crop, sugarcane exhibits a lengthy growth cycle and a sustained root system, establishing a long-lasting rhizosphere and a reliable source of nutrients for its bacterial community, which typically results in a more stable symbiotic interaction (Tayyab et al., 2022). Plant growth-promoting bacteria (PGPB) enhance the chemical properties of the rhizosphere soil and enzyme activity, improve the efficiency of nitrogen utilization in sugarcane, and facilitate water conservation by effectively regulating stomatal closure (Pereira et al., 2019). This management of stomata helps maintain leaf water potential and relative water content (Wei et al., 2013; Aguiar et al., 2016; Wang et al., 2023c) (**Table 1**). Current research has predominantly focused on the drought resistance of sugarcane, examining physiological and biochemical impacts, morphological changes in the rhizosphere and leaves, transgenic breeding, and cultivation practices (Liu et al., 2022a). Nonetheless, a significant gap persists in systematic research that integrates the role of root system bacterial communities in conferring drought resistance to sugarcane. Plants can modulate the activity of soil microorganisms under diverse drought conditions by altering the constituents and profiles of their root exudates (Hassan et al., 2019; Williams and de Vries, 2020). However, much of our understanding of plant–PGPB interactions under drought comes from noncrop species, with crop species potentially selected for traits that may inherently reduce drought tolerance and beneficial interactions with the rhizosphere microbiota (de Vries et al., 2020). In summary, we investigated the adaptive capacities of sugarcane rhizosphere bacterial communities under diverse drought stress scenarios, aiming to enhance our understanding of the mechanisms governing sugarcane–rhizosphere bacterial interactions during drought. We synthesized the effects of multiomics factors, such as sugarcane root architecture, soil metabolites, and nutrients, on rhizosphere bacterial community structure under different levels of drought stress. This exploration holds significant potential, offering novel pathways to enhance our understanding of these interactions and proposing avenues to deepen our insight into the interplay between sugarcane and rhizosphere bacteria in response to drought.

## 2 Rhizosphere environmental changes and adaptive responses of bacterial communities in sugarcane under the influence of drought

### 2.1 Rhizosphere soil nutrient changes

Significant alterations in soil nutrient levels substantially affect the rhizosphere environment of sugarcane plants during drought stress. Under such conditions, the total carbon, nitrogen, and phosphorus contents in soil may diminish because of the constraints imposed on microbial activity, which in turn decelerates the decomposition of organic matter and the mineralization of nutrients (Siebert et al., 2019; Gao et al., 2020; Deng et al., 2021). This reduction in nutrient availability directly impairs the ability of sugarcane roots to assimilate nutrients, thereby restricting plant growth and yield (Siebert et al., 2019; Deng et al., 2021). However, the effectiveness of soil nutrients in the sugarcane rhizosphere might not be severely compromised under mild drought conditions. Several studies have indicated that sugarcane initiates the recruitment of specific rhizosphere bacteria by altering root exudates, thereby enhancing its resilience to drought (Liu et al., 2021a, 2021b; Dao et al., 2023; Xing et al., 2023).

This recruitment process is a critical mechanism of adaptation for sugarcane in drought environments and enhances soil nutrient availability. Root exudates serve as nutrient sources for rhizosphere bacteria, fostering their growth and metabolic activities, which may, in turn, improve the availability of nutrients within the soil (Zhao et al., 2021; Balyan and Pandey, 2024). A significant shift in the rhizosphere bacterial community of sugarcane was observed when the field water-holding capacity of the soil fell below a critical threshold of 50%, indicating a transition from a reliance on the effectiveness of soil nutrients to a dependence on the rhizosphere bacterial community to enhance drought tolerance (Dao et al., 2023). At this juncture, sugarcane increasingly relies on microorganisms capable of thriving under drought conditions, including Streptomycetales and Rhizobiales, which may assist in coping with drought by producing plant growth regulators, enhancing soil structure, and improving nutrient uptake capacity (Ebrahimi-Zarandi et al., 2023; Nong et al., 2023; Yang et al., 2024) (**Table 1**). As drought intensifies, nutrient availability in sugarcane rhizosphere soils becomes increasingly critical. Under these circumstances, sugarcane may need to increasingly rely on changes in root secretions to enlist the aid of drought-tolerant rhizosphere bacteria, which can facilitate access to and utilization of less-soluble nutrients in the soil. This adaptation is essential for sustaining plant vitality and productivity during water scarcity.

Changes in soil enzyme activity are key bioindicators of soil nutrient dynamics. The rhizosphere of sugarcane is notably influenced by drought conditions, with changes in soil enzyme activity significantly influencing nutrient cycling and plant growth (Jamir et al., 2019; Bogati and Walczak, 2022). Enzymes such as acid phosphatase, urease, and catalase facilitate the mineralization and transformation of nutrients in the soil, thereby directly affecting the availability of nutrients to sugarcane (Neemisha and Sharma, 2022). In drought environments, reduced soil moisture leads to a decline in enzyme activity, which in turn slows down the decomposition of organic matter and reduces the nutrient supply available to sugarcane, thereby affecting growth and productivity (Liu et al., 2021b). Moreover, soil enzymatic activity is closely linked to the structure and function of the rhizosphere microbial community. Changes in the rhizosphere bacterial community structure under drought conditions can modify the composition and activity of soil enzymes, which then influence nutrient dynamics within the sugarcane rhizosphere (Schimel, 2018; Bogati and Walczak, 2022). Additionally, a reduction in soil enzyme activity during drought may affect plant drought tolerance. Certain soil enzymes participate in the synthesis or degradation of organic substances related to drought resistance, such as proline, which plays a protective role in plants facing water scarcity (Deng et al., 2015). The effect of soil enzyme activity on the rhizosphere environment of sugarcane under drought conditions is multifaceted and encompasses soil nutrient cycling, microbial community structure, plant nutrient uptake, and plant drought resistance. Delving deeper into these processes will enhance our understanding of how drought affects sugarcane production and aid in the formulation of more effective strategies to mitigate its effects.

In conclusion, the effect of drought stress on soil nutrients is characterized by a complex interplay of factors, including a reduction in nutrient content, a decline in nutrient availability, and a decrease in soil enzyme activity. These elements, when combined, significantly influence the growth and health of sugarcane in the rhizosphere. A thorough understanding of these precise shifts in soil nutrient dynamics is imperative for a more nuanced appraisal of the alterations within the sugarcane rhizosphere under drought conditions. Furthermore, it is crucial to understand the adaptive mechanisms of rhizosphere bacterial communities that play a critical role in plant resilience and the overall response to water scarcity. This knowledge will inform the development of more precise and effective strategies to enhance the sustainability and productivity of sugarcane cultivation in drought environments.

### 2.2 Rhizosphere soil metabolite composition

Within the sugarcane rhizosphere, the sources of soil metabolites are multifaceted. The formation of these metabolites results not only from secretions by the sugarcane root system but also from the metabolic processes of rhizosphere microorganisms (Ponomarova et al., 2017). Marked variations characterize the composition and content of rhizosphere soil metabolites in sugarcane subjected to varying degrees of stress (Zhao et al., 2020). The types and contents of soil metabolites (fatty acids, carboxylic acids, carbohydrates, and amino acids) in sugarcane increased significantly under mild drought conditions, while long-chain organic acids (oleic acid and linoleic acid) increased under moderate drought conditions, and long-chain organic acids (oleic acid, monopalmitin, and monoolein) increased under severe drought conditions, compared to well-watered conditions (Xing et al., 2023). These metabolic changes are associated with the selective enrichment of specific bacterial communities within the sugarcane rhizosphere under drought stress. This enrichment is believed to act as an adaptive mechanism that enhances plant drought resilience (Cesari et al., 2019; Woo et al., 2020). As drought severity progresses, carbon uptake by sugarcane decreases, concurrently diminishing carbon flux in rhizosphere exudates. This reduction restricts interactions between sugarcane and its associated rhizosphere bacteria (Ingrisch et al., 2018; Karlowsky et al., 2018). Since rhizosphere bacteria rely on organic exudates from the sugarcane root system for their carbon supply, their activities may be concurrently constrained by carbon scarcity and water scarcity under severe drought conditions (Dijkstra et al., 2013). In conclusion, although drought stress can hinder the physiological metabolism of sugarcane, this species exhibits resilience by adjusting its photosynthetic allocation, metabolic pathways, and production of osmotic protectants. These adaptive responses reflect the intrinsic physiological mechanisms of the plant, which are further influenced by its interactions with root-associated bacterial communities. Through their metabolic activities and symbiotic relationships, these communities support the strategies plant’s strategies for adapting and thriving under drought conditions. They aid in nutrient acquisition, boost stress tolerance, and improve water-use efficiency, thereby reinforcing the overall response of plants to drought.

## 3 Changes in the rhizosphere bacterial community of sugarcane under drought stress

### 3.1 Influencing factors and adaptability of sugarcane rhizosphere bacterial community diversity

The diversity and composition of sugarcane rhizosphere bacterial communities are intricately shaped by a dynamic equilibrium, influenced by various factors. Under drought stress, soil metabolites, soil nutrients, sugarcane genotypes, and root system traits have emerged as pivotal determinants of rhizosphere bacterial community structure (Zhao et al., 2020; Liu et al., 2021a, 2021b; Dao et al., 2023; Xing et al., 2023). Under drought conditions, a general decline in the diversity of sugarcane rhizosphere soil bacteria has been observed. However, the specific impacts of varying drought intensities reveal a complex and variable response to water stress (Liu et al., 2021a; Xing et al., 2023). Under mild drought conditions, sugarcane and its associated rhizosphere bacterial communities exhibit a degree of tolerance. Adaptive alterations in root exudates, particularly increases in organic acids, sugars, and amino acids, may lead to increased bacterial diversity (Zhalnina et al., 2018; Chen et al., 2022). However, under moderate to severe drought conditions, a significant reduction in the alpha diversity of sugarcane rhizosphere bacterial communities has been observed, suggesting that the stress may have surpassed the tolerance limits of certain bacterial species, resulting in pronounced shifts in community composition (Xing et al., 2023). Streptomycetales and Rhizobiales bacteria have been shown to play a crucial role in the response to drought stress in the sugarcane rhizosphere (Liu et al., 2021a; Dao et al., 2023). These bacterial strains become more abundant under drought stress and are positively associated with key sugarcane root characteristics, including the number of root tips and total root length. This finding suggests that these strains may play a vital role in promoting the development of sugarcane roots and enhancing the efficiency of water and nutrient uptake (Maia Júnior et al., 2019; Liu et al., 2021b; Xing et al., 2023). In addition, soil nutrient status, particularly the levels of available phosphorus (P) and soil acid phosphatase activity, is correlated with the alpha diversity of sugarcane rhizosphere bacterial communities, underscoring the regulatory role of soil nutrients in shaping bacterial diversity (Zhao et al., 2020; Liu et al., 2021a, 2021b). Comparative analyses across sugarcane varieties have revealed that drought-tolerant varieties harbor a higher abundance of drought-associated bacteria, even under non-drought conditions, whereas these bacterial populations only increase under stress conditions in drought-sensitive varieties. Furthermore, significant differences in rhizosphere soil nutrient composition and enzyme activities have been observed among drought-tolerant varieties, highlighting the close relationship between sugarcane drought tolerance and the adaptive capabilities of rhizosphere bacterial communities (Zhao et al., 2020; Liu et al., 2021b; Dao et al., 2023). The diversity of sugarcane rhizosphere bacterial communities therefore represents a complex ecosystem influenced by multiple factors. A thorough understanding of the interplay between these factors and the establishment and function of rhizosphere bacterial communities is essential to guide the development of drought-tolerant sugarcane varieties, optimize agricultural practices, and enhance sugarcane yield and quality. Comprehensive analyses of soil metabolites, soil nutrients, sugarcane genotypes, and root characteristics can offer a more holistic understanding of the adaptive mechanisms of rhizosphere bacterial communities, thereby providing evidence to inform the sustainable cultivation of sugarcane under drought conditions.

### 3.2 Effect of Streptomycetales on the drought performance of sugarcane

**Figure 2:**
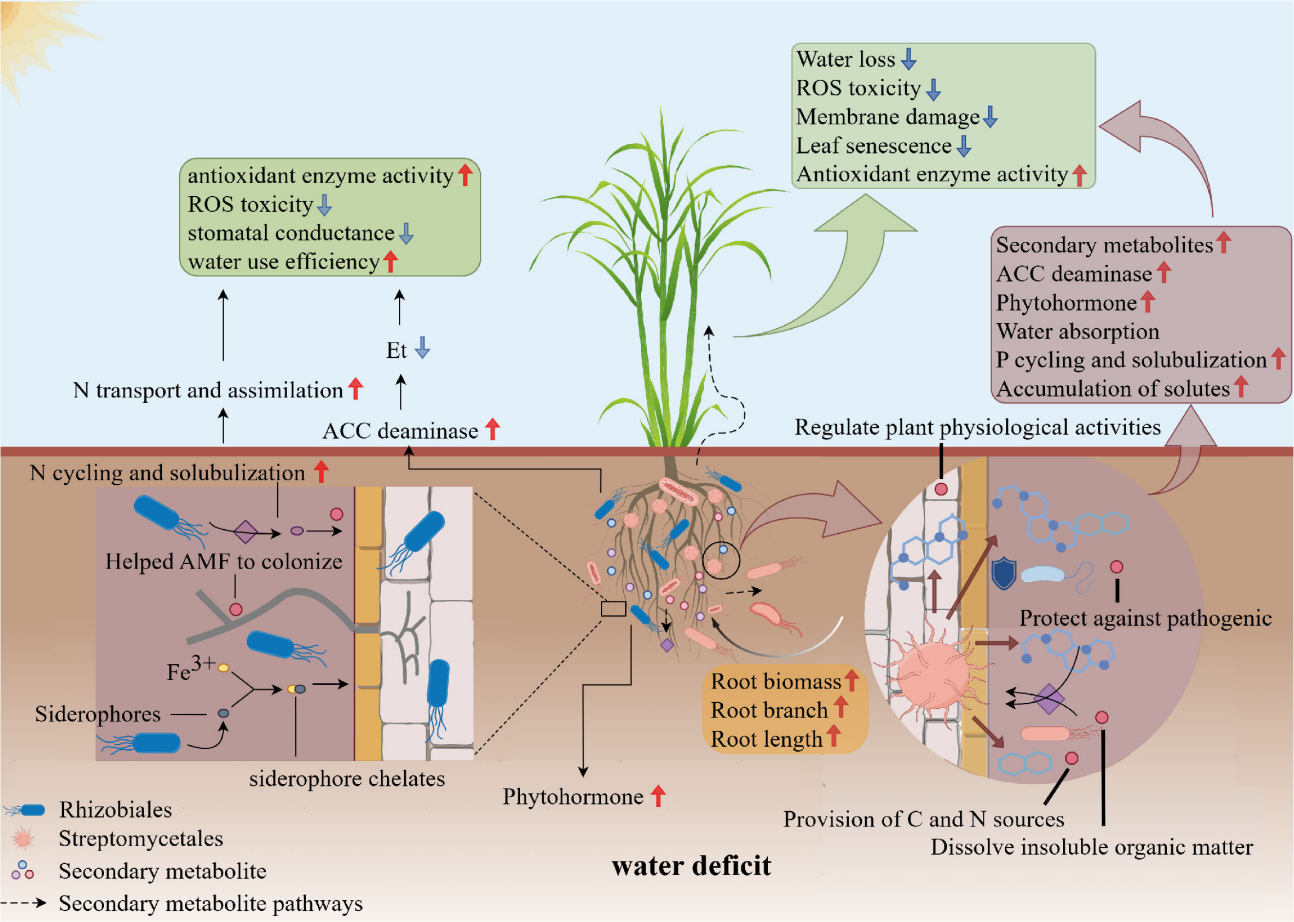
Effects of Streptomycetales and Rhizobiales on drought tolerance of sugarcane under drought conditions. The abundance of Streptomycetales and Rhizobiales increased in the inter-root of sugarcane under drought conditions. Streptomycetales can secrete a variety of secondary metabolites, which can enhance the drought resistance of sugarcane by protecting against pathogens, dissolving insoluble organic matter to improve plant root morphology, and enhancing root water and nutrient uptake, thereby improving drought tolerance. Rhizobiales, on the other hand, form a symbiotic relationship with the sugarcane root system, fixing atmospheric nitrogen and providing an essential nitrogen source. This symbiotic relationship enhances the growth and survival of sugarcane under drought conditions. Additionally, Rhizobiales can produce a variety of phytohormones that regulate physiological responses in sugarcane. Streptomycetales and Rhizobiales work together through different mechanisms to enhance the drought resistance of sugarcane under drought conditions.

Streptomycetales is the bacterial strain most responsive to drought stress in sugarcane and the relative abundance of this bacterial strain increases significantly under moderate and severe drought conditions (Liu et al., 2021a; Xing et al., 2023). Xu et al. (2018) reported a significant correlation between the relative abundance of Streptomycetales in plant root systems and host drought resistance. The mycelial and spore-forming properties of Streptomycetales enable these bacteria to survive in harsh environmental conditions compared to other PGPBs (Vurukonda et al., 2018). Streptomycetales can also produce many secondary metabolites and volatile metabolites (Olanrewaju and Babalola, 2019), and these metabolites can alter the structure of the rhizosphere bacterial community and influence crop root conformation (Chukwuneme et al., 2020; Zhang et al., 2024) (**Figure 2**).

Streptomycetales is a group of Actinobacteria belonging to the order Actinomycetales. These bacteria thrive in less hydrated soil environments and readily metabolize recalcitrant carbon sources typically found in such soils (Ebrahimi-Zarandi et al., 2023). Actinomycetes have also been associated with an inhibitory effect on soil fungal pathogen infestation, which increases with drought stress (Varela et al., 2016; Fallah et al., 2023). The survival of Streptomycetales under drought conditions can be attributed to its characteristic high substrate affinity coupled with a relatively slow growth rate compared to other sugarcane rhizosphere bacteria, enabling it to persist under limited moisture conditions (Acosta-Martinez et al., 2014; Nazari et al., 2023). Gram-positive bacteria, including Streptomycetales, are particularly adept at harnessing a diverse range of carbon sources more effectively than their Gram-negative counterparts. They possess the metabolic versatility to assimilate inorganic N from nutrient-poor, drought-affected soils, thereby facilitating the enzymatic breakdown of organic matter. In contrast, Gram-negative bacteria, such as Aspergillus and Acidobacteria, are primarily reliant on the immediate availability of carbon and N from root exudates (Reinsch et al., 2014; Martiny et al., 2017). Studies have indicated a positive correlation between drought severity and the presence of genes encoding carbohydrases in actinomycetes (Zhalnina et al., 2018). This genetic predisposition may underlie the increased carbohydrate content of soil metabolites, potentially enhancing the attraction and colonization of Streptomycetales in the rhizosphere of sugarcane, particularly during periods of prolonged drought stress.

Streptomycetales are crucial in the rhizosphere of sugarcane for maintaining plant health and enhancing drought resilience. Drought resistance is achieved through exudation of 1-aminocyclopropane-1-carboxylate (ACC) deaminase and secondary metabolites, such as antibiotics and indole-3-acetic acid (IAA) (Barnawal et al., 2013; Kour et al., 2024). The metabolites produced by Streptomycetales, such as antibiotics, vitamins, amino acids, and extracellular hydrolases, counter nutrient limitations arising from reduced enzymatic activity during drought, thereby stimulating enhanced nutrient absorption by sugarcane roots (Selim et al., 2019; Chaiharn et al., 2020; Borah et al., 2022). Warrad et al. (2020) observed that a cell-free extract (F2.7) of *Streptomyces* sp. Ac3 significantly increased the levels of total soluble sugars, proline, and betaine in maize, thereby improving its growth and quality under water-scarce conditions. Streptomycetales elevates proline levels by producing ACC deaminase, which aids in the stabilization of subcellular structures, maintenance of cellular redox balance, and mitigation of osmotic stress (Wang et al., 2018b; Chandra et al., 2019; Kour et al., 2022). Streptomycetales contribute to the stability of bacterial diversity of the rhizosphere under severe drought conditions by solubilizing complex compounds and providing carbon and N sources for other bacteria (Viaene et al., 2016; Wang et al., 2018a). Drought affects the availability of effective P in the rhizosphere soil by impeding the decomposition of organic P and the release of inorganic P (Fan et al., 2021). However, Streptomycetales mobilizes soil phosphatases to accelerate the degradation of organic P substrates and enhance phosphate production, thereby supplying plants with essential P (Martín and Liras, 2021). During periods of drought, an increase in soil coverage by the crop root system is associated with more fine roots, thereby enhancing accessibility to essential resources (Gargallo-Garriga et al., 2018). Xing et al. (2023) reported a significant association between the relative abundance of Streptomycetales in the rhizosphere bacterial community and root phenotypes. This association is linked to the ability of Streptomycetales to produce IAA, an organic compound that fosters the proliferation of lateral roots and root hairs. The enhanced development of the root system helps sugarcane locate supplementary water resources in drought environments (Yandigeri et al., 2012; Kurepa and Smalle, 2022; Etesami and Glick, 2024). In addition, the abundance of Streptomycetales is positively associated with the abundance of other rhizosphere bacteria, including Streptosporangiales, Jiangellales, Micrococcales, and Rhizobiales (Xing et al., 2023). Streptomycetales may, therefore, enhance the role of other rhizosphere bacteria in promoting sugarcane growth and drought resistance by producing metabolites that mediate interbacterial communication.

### 3.3 Effect of Rhizobiales on the drought performance of sugarcane

The abundance of Rhizobiales in the sugarcane rhizosphere decreases under mild drought conditions but increases as the severity of drought increases (Xing et al., 2023). In addition to their essential role in promoting plant growth, Rhizobiales can trigger a range of physiological and biochemical stress responses in sugarcane plants. Specifically, PGPB enhance the production of osmoprotectants, which are crucial for maintaining cellular integrity during drought stress. Rhizobiales activate antioxidant enzymes that neutralize the harmful effects of reactive oxygen species, thereby bolstering sugarcane resistance to drought stress (Ahemad and Khan, 2012; Wang et al., 2018a).

Rhizobiales are highly abundant in the sugarcane rhizosphere. During drought events, alterations in soil physicochemical properties and root exudates can create inhospitable conditions for some members of the Rhizobiales community (Liu et al., 2013; Dao et al., 2023). Nonetheless, certain Rhizobiales are adept at capitalizing on sugarcane rhizosphere exudates, thereby ameliorating the rhizosphere environment under drought stress. These adaptable rhizobiales may even dominate the bacterial community in the sugarcane rhizosphere (Gopalakrishnan et al., 2015; Trivedi et al., 2021; del Carmen Orozco-Mosqueda et al., 2022). This capability is attributed to the fact that, as part of the Alphaproteobacteria, Rhizobiales can efficiently metabolize the carbon found in sugarcane rhizosphere exudates (Da Costa et al., 2018). Furthermore, the abundance of *Streptomyces* is positively correlated with that of Rhizobiales (Xing et al., 2023). We propose that Rhizobiales can exploit metabolites from *Streptomyces* and synergistically interact with them to ameliorate soil physicochemical properties and regulate the physiological and biochemical responses of sugarcane.

Under drought conditions, Rhizobiales augment the nutrient response and enhance the drought tolerance of sugarcane through multiple mechanisms (**Figure 2**). Although Rhizobiales are recognized as plant growth-promoting bacteria and for their contribution to stress tolerance, there is a relative scarcity of literature on their specific impact on the drought resilience of non-leguminous plants, such as sugarcane. These bacteria can ameliorate the detrimental effects of drought on plants by modulating leaf stomatal conductance, photosynthetic capacity, and root morphology in non-leguminous species (Moutia et al., 2010). For instance, Chen et al. (2024) demonstrated that inoculation with *Rhizobium rhizogenes* led to an elevation in endogenous abscisic acid (ABA) levels in *Arabidopsis*, bolstering the sensitivity and adaptive response of the plant to drought stress. N and P are acknowledged as significant limiting factors in agroecosystems (Zuluaga and Sonnante, 2019; Bihari et al., 2022), and the acquisition of these elements by plants is closely connected with the availability of water. N must be dissolved in water to be absorbed by plant roots (Araus et al., 2020). During periods of drought, water accessibility is compromised, restricting the uptake of various nutrients (Osakabe et al., 2014). Furthermore, numerous genes involved in N transport and assimilation are suppressed at the transcriptional level under drought stress (Ristova et al., 2016). It has been observed that *Arabidopsis thaliana* employs a common set of genes to respond to N and drought signals (Cerda and Alvarez, 2024). Although Rhizobiales are often associated with legumes, providing N to their host plants through symbiosis, they have also been reported to replenish soil N in a saprophytic state and function as endophytes, thereby increasing the uptake of essential mineral elements such as N and P in sugarcane (Li Ting et al., 2013; Fallah et al., 2023). Conversely, the regulation of water channel proteins by different N levels influences hydraulic conductance (Araus et al., 2020). The availability of N in the soil facilitates water entry into cellular spaces by affecting the hydraulic properties of the cell membrane and intracellular nitrate concentration (Zheng et al., 2018). N availability also plays a role in stomatal regulation, affecting transpiration and water-use efficiency (Matimati et al., 2014). Rhizobiales may contribute to the demineralization of soil organic matter by modulating the activity of extracellular enzymes involved in soil nutrient cycling. This process increases total N and available P contents in the rhizosphere soil, creating conditions conducive to plant growth under drought stress (Ahluwalia et al., 2021; Wang et al., 2023b). Elevated levels of available P can effectively mitigate P stress caused by an imbalanced soil nitrogen-phosphorus ratio, promoting the growth of underground plant parts (Huang et al., 2018; Liu et al., 2021b). In addition, Rhizobiales produce phytohormones, including IAA and abscisic acid, which regulate root conformation in sugarcane and facilitate nutrient exploration (Barquero et al., 2022; Lopes et al., 2023).

### 3.4 Effect of other rhizosphere bacteria on drought performance of sugarcane

The rhizosphere bacterial community composition is generally consistent across different sugarcane varieties, but it differs under drought stress. For example, in the Zhongzhe 1 sugarcane variety, the bacteria Streptosporangiales and Sphingomonadales were enriched under drought conditions (Dao et al., 2023). Streptosporangiales, an order within Actinomycetes, has been observed to increase in abundance under drought conditions. However, there is a lack of research substantiating their growth-promoting effects on plants, specifically sugarcane, and their roles in enhancing drought tolerance. Conversely, Sphingomonadales are recognized for their superior adaptation to arid soils as oligotrophic bacteria and their proficiency in degrading organic pollutants (Roy et al., 2012; Kunihiro et al., 2013; Luo et al., 2019). *Sphingomonas* has been identified as a PGPB that emits volatile organic compounds (VOCs), which stimulate plant growth and mitigate the effects of plant pathogens, thereby improving drought resistance (Farag et al., 2013; Luo et al., 2020; Poudel et al., 2021). *Gluconacetobacter diazotrophicus* PAL5, an endophytic nitrogen-fixing bacterium associated with sugarcane, enhances the drought tolerance of sugarcane (Vargas et al., 2014). Nong et al. (2023) demonstrated upregulation of the biosynthesis of carotenoids and the terpenoid backbone by *Burkholderia* in sugarcane. The Guangxi sugarcane variety GT42D has been shown to harbor Burkholderiaceae as its core flora under drought stress (Liu et al., 2021a) (**Table 1**). Despite these findings, there is a scarcity of studies investigating the growth promotion and drought resistance properties of *Sphingomonas*, *Burkholderia*, and *Gluconacetobacter* in sugarcane. Further research is required to understand the mechanisms of action and to explore the potential applications of these bacteria in agriculture. Of note, *Bacillus* species are widely distributed across dynamic agroecosystems due to their robust tolerance to stress, which is facilitated by spore formation (Vardharajula et al., 2011; Radhakrishnan et al., 2017; Gowtham et al., 2020). *Bacillus* species have also been shown to enhance the drought tolerance of sugarcane (Chandra et al., 2018; Fonseca et al., 2022), even under severe drought stress. Almeida et al. (2024) demonstrated that inoculation with *Bacillus licheniformis* and *Bacillus subtilis* significantly enhanced the survival of presprouted sugarcane seedlings under water-limited conditions. These bacteria also improved the water-use efficiency of presprouted seedlings under severe drought conditions by nearly 200%. Furthermore, the abundance of *Bacillus* in sugarcane appears to remain stable under mild and moderate drought conditions and may decrease under severe stress. These observations highlight the potentially important role of *Bacillus* as a bioregulator for improving crop drought tolerance. Nevertheless, the variation in *Bacillus* abundance under different drought intensities may be influenced by a multitude of ecological factors and plant–microbe interactions. Furthermore, in-depth studies are needed to fully understand the mechanism of its action in dynamic agroecosystems.

## 4 Conclusions

Sugarcane, a vital cash and energy crop worldwide, is acutely sensitive to water deficit throughout the growth cycle. Rhizosphere bacterial communities are crucial for crop growth and development. Our review evaluated the abundance of rhizosphere bacterial communities under crop water stress and examined evidence of plant–bacteria dynamics. Diversity within the bacterial community in the rhizosphere soil of sugarcane significantly changes when soil water-holding capacity reaches 50%. The abundance of some bacterial groups diminishes as the severity of drought stress increases, whereas the abundance of Streptomycetales and Rhizobiales increases with increasing drought stress. Streptomycetales enhance drought resistance in sugarcane by producing various secondary metabolites. They also support nutrient availability in the rhizosphere by supplying carbon or nitrogen to other microbes, thereby improving soil fertility. Rhizobiales, on the other hand, support nitrogen acquisition in sugarcane and produce siderophores to assist with iron uptake under drought conditions. There are differences in the response of bacterial rhizosphere communities to drought stress across sugarcane varieties. For example, during drought stress, Burkholderiaceae was prominent in the rhizosphere bacterial communities of the Guangxi sugarcane variety GT42D. Despite considerable advances in knowledge, our understanding of the interactions between PGPB and sugarcane under drought stress requires further investigation. Framework modeling of sugarcane rhizosphere bacterial communities and their productivity under varying levels of drought stress could provide important theoretical support and guidance for sugarcane cultivation and production practices.
